# Context matters in understanding the vulnerability of women: perspectives from southwestern Uganda

**DOI:** 10.1186/s13690-020-00523-x

**Published:** 2021-01-07

**Authors:** Neema Murembe, Teddy Kyomuhangi, Kimberly Manalili, Florence Beinempaka, Primrose Nakazibwe, Clare Kyokushaba, Basil Tibanyendera, Jennifer L. Brenner, Eleanor Turyakira

**Affiliations:** 1 Department of Human Development and Relational Sciences, Faculty of Interdisciplinary Studies, Mbarara University of Science and Technology, Mbarara, Uganda; 2 Healthy Child Uganda, Maternal Newborn and Child Health Institute, Mbarara University of Science and Technology, Mbarara, Uganda; 3 Department of Community Health Sciences, Cumming School of Medicine, University of Calgary, Calgary, Canada; 4 Department of Nursing, Faculty of Medicine, Mbarara University of Science and Technology, Mbarara, Uganda; 5 Department of Education Foundations, Faculty of Education, University of Saint Joseph Mbarara, Mbarara, Uganda; 6 Departments of Pediatrics and Community Health Sciences, Cumming School of Medicine, University of Calgary, Calgary, Canada; 7 Department of Community Health, Faculty of Medicine, Mbarara University of Science and Technology, Mbarara, Uganda

**Keywords:** Vulnerability, MNCH programming, Diversity, MNCH interventions, Community engagement, Context matters, Women

## Abstract

**Background:**

Vulnerability at the individual, family, community or organization level affects access and utilization of health services, and is a key consideration for health equity. Several frameworks have been used to explore the concept of vulnerability and identified demographics including ethnicity, economic class, level of education, and geographical location. While the magnitude of vulnerable populations is not clearly documented and understood, specific indicators, such as extreme poverty, show that vulnerability among women is pervasive. Women in low and middle-income countries often do not control economic resources and are culturally disadvantaged, which exacerbates other vulnerabilities they experience. In this commentary, we explore the different understandings of vulnerability and the importance of engaging communities in defining vulnerability for research, as well as for programming and provision of maternal newborn and child health (MNCH) services.

**Methodology:**

In a recent community-based qualitative study, we examined the healthcare utilization experiences of vulnerable women with MNCH services in rural southwestern Uganda. Focus group discussions were conducted with community leaders and community health workers in two districts of Southwestern Uganda. In addition, we did individual interviews with women living in extreme poverty and having other conventional vulnerability characteristics.

**Findings and discussion:**

We found that the traditional criteria of vulnerability were insufficient to identify categories of vulnerable women to target in the context of MNCH programming and service provision in resource-limited settings. Through our engagement with communities and through the narratives of the people we interviewed, we obtained insight into how nuanced vulnerability can be, and how important it is to ground definitions of vulnerability within the specific context. We identified additional aspects of vulnerability through this study, including: women who suffer from alcoholism or have husbands with alcoholism, women with a history of home births, women that have given birth only to girls, and those living on fishing sites.

**Conclusion:**

Engaging communities in defining vulnerability is critical for the effective design, implementation and monitoring of MNCH programs, as it ensures these services are reaching those who are most in need.

## Background

In the context of healthcare, vulnerability affects access to and utilization of health services including Maternal, Newborn, and Child Health (MNCH) services [4]. While the global magnitude of vulnerable women has not been clearly documented, this population is noted to be substantial, especially in low-income countries [7]. Different frameworks have been used to define vulnerable women. Conventionally, such women have been identified based on income falling below the acceptable benchmark of welfare, along with other demographic characteristics such as ethnicity, education level, and locale - rural versus urban status [2, 8]. Culture and the ability to make decisions can also have an impact on vulnerability as women in many low-resource settings do not control economic resources, which further exacerbates their vulnerability [6].

Among donors, non-governmental organizations and governments themselves, there is growing interest in targeting MNCH services to vulnerable women who may otherwise not access these services. Using the conventional vulnerability assessment “checklists” based on the factors noted above for programming decisions can lead to the identification of many vulnerable women in low-income countries [5]. Within restricted program budgets, stringent thresholds are often set to identify a preset target number of eligible beneficiaries. However, limitations of targeting mechanisms based on statistical modelling of national household survey datasets, commonly used to identify the eligible poor for social assistance programs, have been highlighted [3]. For example, the implementation of poverty reduction programs in low and middle income countries, coupled with the transition in certain aspects of lifestyle (e.g. changes in housing structures), mean that manifestations of vulnerability continue to shift with some categories of people becoming more complex to identify using conventional criteria. Indicators of poverty used in scoring individuals or household status, such as living in a grass-thatched dwelling, become rare to find and irrelevant in some rural communities despite chronic poverty. Moreover, factors that are not traditionally considered socio-economic in nature are not considered. Consequently, some women who are in need of assistance are left out when their estimated poverty score is lower than the set threshold despite other unusual factors which prevent them from accessing MNCH services. There are incredible implications for health equity when those who are most in need of services are not reached. In this commentary we explore community perceptions about women vulnerability in comparison with conventional criteria, and the implications for research and targeted programming.

## Methodology

During a recent community-based qualitative study, we examined the utilization experiences with MNCH services in rural southwestern Uganda, which were implemented as part of an intervention by Healthy Child Uganda (2012–2015). We initially developed participant selection criteria to purposively identify vulnerable women based on the conventional economic and demographic characteristics. Vulnerable women eligible to participate in our study were those in extreme poverty and with at least one of the following additional characteristics: (a) being an adolescent mother, (b) having many children with poor spacing, (c) physical disability of the woman or her husband, (d) living with HIV [8]. As part of our recruitment strategy, we collaborated with local community health workers (CHWs), who helped us identify vulnerable women in their communities. For example, women living in extreme poverty were identified by CHWs based on local perceptions of what constitutes extreme poverty for community families. In addition, we enrolled community leaders and CHWs to take part in focus group discussions and share their perspectives on vulnerability, barriers to and facilitators of women’s access to MNCH services in their communities. All interviews and focus group discussions took place with the explicit written consent of participants, which was obtained by reading and explaining the consent form before it was signed by all participants. Participants who were unable to read and write expressed their consent with the thumbprint in the presence of their CHW (appointed community representative) as a witness. Important aspects of the consent form included confidentiality, right to participate and withdraw, benefits and risks of taking part in the study. Participant consent was obtained in the local language. Participants were informed of the importance of voice recording and those not willing to have their voices recorded during the interview would not be enrolled in the study. In-depth interviews took place in a quiet environment with no disturbances and where no intruders could disrupt the conversations.

## Findings and discussion

Through the narratives of community leaders and vulnerable women, we identified additional aspects of vulnerability beyond categories suggested by conventional methodology and the research team. The community’s own definitions of ‘vulnerable’ were more nuanced and specific to their context. Communities felt these categories could be recognized within their settings; community members could identify who was vulnerable and who was not, based on their context. For instance, women that they considered “vulnerable” were those who were neglected by their partners and/or family members often due to underlying causes, including giving birth to girls only and therefore, providing no heir to the husband. Vulnerable women were also those who consumed a lot of alcohol, and thus, affecting their ability to live responsibly, or who lived with partners that are alcoholic. Other aspects of vulnerability also included women with a history of healthy home births, and women living in fishing villages away from their larger extended family support. The transient nature of the male population that is common in fishing villages exacerbates the vulnerability of married women in these areas. Some of the ways they described “vulnerability” were surprising to us (e.g. women with healthy home births), and indeed required a closer look at the women’s experiences through their stories, in order to understand how vulnerability can manifest. A history of successful home births overshadows potential risk of childbirth complications in subsequent pregnancies. Additionally, it is interesting to note that women that we considered “vulnerable” did not consider themselves vulnerable – they described their resilience and the protective factors that allowed them to access MNCH services, despite poverty, particularly their family or husband’s support.

It becomes apparent that the more nuanced community perspectives on what constitutes “vulnerability” in their context can allow researchers and healthcare systems to better identify women who could benefit from targeted programming. Recognizing this diversity of vulnerability is of high importance in designing, implementing, growing and garnering success for MNCH programs. A lack of careful consideration of the contextual and nuanced factors related to vulnerability means some vulnerable women may be overlooked. Moreover, there is potential for devaluing community knowledge, which can affect community support and ownership for the intervention, that is essential for program sustainability.

In defining vulnerability, community engagement is crucial. Community engagement also leads to improved and greater outcomes by building trust, supporting better individual decision making, growing community satisfaction with the performance of organizations, and garnering ownership and sustainability of the organization’s interventions [1]. It reveals more nuanced and less documented factors that hinder women from utilizing specific health services. Some development partners and program implementers may argue that bottom-up approaches slow the scaling up of programs that already have a record of delivering positive results. However, without bottom-up approaches, many initiatives fail to reach those who would benefit most and/or cannot be sustained over time due to weak relationships that failed to be developed during initial program development [1]. Not asking communities what they want, nor recognizing and honouring their experiences undermines trust. Failing to engage communities in defining who the vulnerable women are will hinder the effectiveness of MNCH interventions aimed at these women.

Vulnerability is context dependent and the involvement of community members who understand their context does aid formulation of criteria of vulnerability using characteristics that are easily recognised by them and ensures that the most vulnerable women are reached. Policymakers and other leaders should engage community members as active partners, utilizing their deep experiences in their communities to enhance the potential for program success. When communities are engaged, they develop the leadership necessary to demand services and accountability. This generates the support necessary for long-term change and encourages beneficiaries to go beyond merely participating, but to also become champions for MNCH programs. We have learned that in our setting, using only a conventional ‘demographics-based’ vulnerability definition during community program planning will deprive our communities of the participatory opportunity to really voice their needs with regard to where resources should be allocated to reach those who need services most. Failing to engage communities in defining who is most vulnerable diminishes potential impact, undermines community trust, and compromises program sustainability.

## Conclusion

Partners, donors and governments who continue to depend only on conventional published definitions of ‘vulnerability’ will miss opportunities for maximum program value to address inequities that persist. The addition of community-derived definitions and perspectives will strengthen intervention effectiveness. Although our experience is in a low-income country in a particular setting, we suspect that similar findings on the diversity of vulnerability will be found in middle- and high-income countries if communities are consulted. To reach the women who are most vulnerable, it is time to listen to the voices of our communities. It is only through meaningful engagement with communities that we will be able to achieve the needed improvements in the health of mothers and children globally.

## Data Availability

The datasets used and/or analysed during the current study are available from the corresponding author on reasonable request.

## Abbreviations

CHWs: Community Health Workers
HIV: Human Immunodeficiency Virus
MNCH: Maternal Newborn and Child Health
